# Oncofetal reprogramming and cellular plasticity in colorectal cancer: from mechanisms to clinical translation

**DOI:** 10.3389/fonc.2026.1912866

**Published:** 2026-08-25

**Authors:** Haoyu Wang, Songhao Liu, Mingxuan Zhang, Chunping Deng, Zhenguo Han

**Affiliations:** Third Hospital of Shanxi Medical University, Shanxi Bethune Hospital, Shanxi Academy of Medical Sciences, Tongji Shanxi Hospital, Taiyuan, China

**Keywords:** AP-1, cellular plasticity, colorectal cancer, drug tolerance, epithelial–mesenchymal plasticity, oncofetal reprogramming, tumor microenvironment, YAP/TAZ

## Abstract

Colorectal cancer (CRC) progression and metastasis, as well as tolerance to therapy, are driven not only by genetic alterations but also by cell-state plasticity. Oncofetal reprogramming (OnF) refers to the reactivation of fetal intestinal developmental or injury-repair programs in tumor cells, thereby weakening adult lineage identity and enhancing the capacity for state transitions. Available evidence suggests that, in specific Wnt-dependent or APC-aberrant contexts, YAP/TAZ–TEAD and AP-1 may act cooperatively to establish and maintain the OnF state, whereas Wnt, EGFR–MAPK, FGF/FGFR, TGF-β, and extracellular matrix signaling contribute to its establishment and maintenance in a context-dependent manner. The OnF state can also intersect with epithelial–mesenchymal plasticity, dynamic stemness, drug-tolerant persister states, and immune and stromal niches, collectively increasing cellular heterogeneity, adaptability to therapy, and relapse potential in CRC. This review discusses the conceptual boundaries and operational criteria for identifying OnF in CRC, examines its regulatory mechanisms, plasticity-associated phenotypes, and translational relevance, and emphasizes the importance of distinguishing direct evidence of OnF from evidence of related plasticity mechanisms. Further elucidation of the OnF state may facilitate biomarker development and anti-plasticity therapies, although its clinical translation will require standardized state classification and clinical validation.

## 1 Introduction

Colorectal cancer (CRC) ranks among the leading malignancies worldwide in terms of both incidence and mortality (1). Despite continued advances in surgery, chemotherapy, targeted therapy, and immunotherapy, metastasis, recurrence, and treatment resistance remain the principal causes of death among patients with CRC (2, 3). A growing body of evidence indicates that evolutionary models based solely on the linear accumulation of genetic mutations cannot fully account for the dynamic changes involved in CRC metastasis, therapeutic adaptation, and the emergence of intratumoral heterogeneity (4).

**Figure 1:**
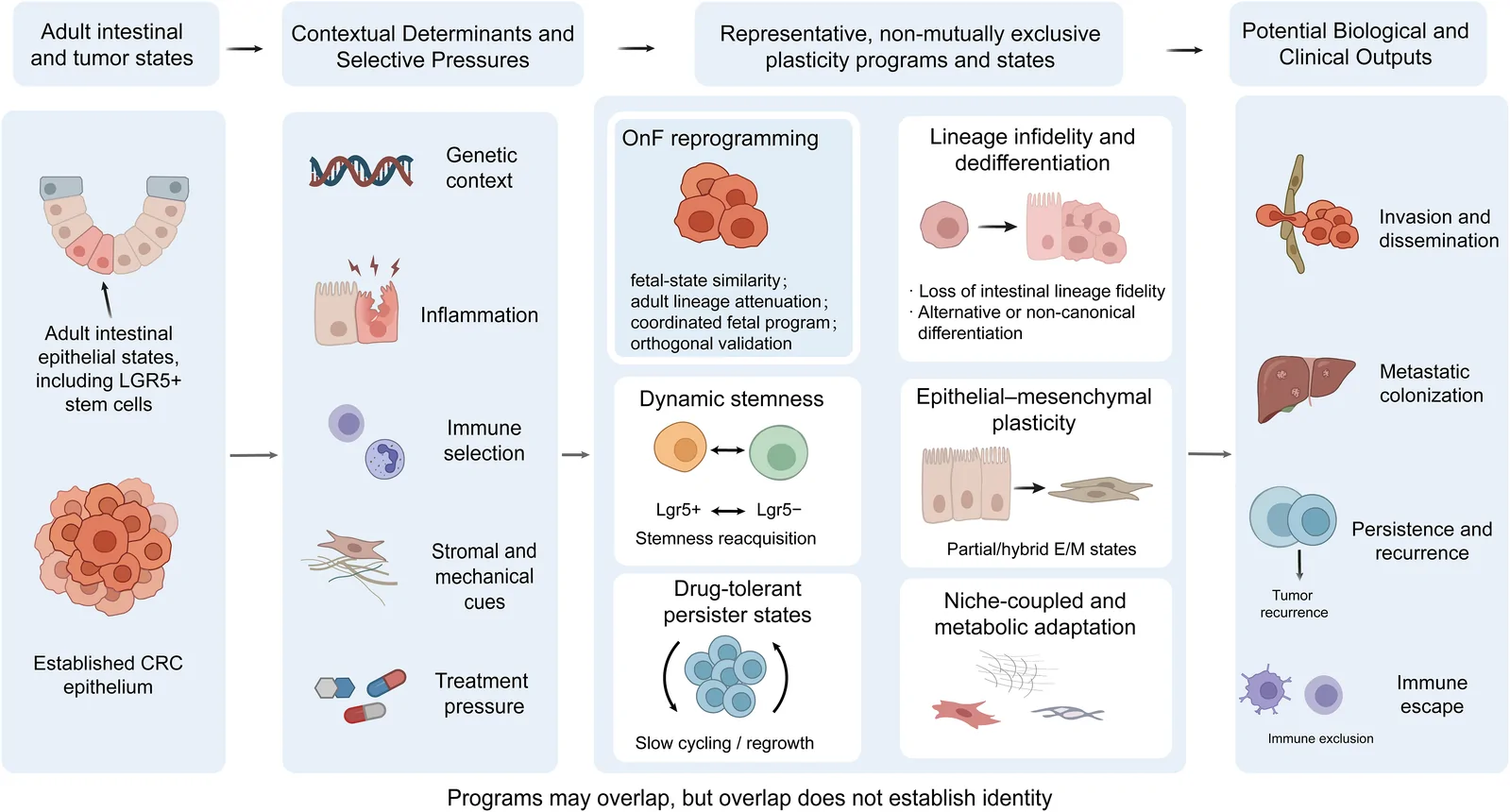
Context-dependent cellular plasticity programs in colorectal cancer. Adult intestinal epithelial cells and established CRC epithelial states are jointly shaped by genetic background, inflammatory and immune pressures, stromal and mechanical cues, and therapeutic exposure. These inputs can promote overlapping but non-equivalent plasticity programs, including oncofetal reprogramming, lineage infidelity, dynamic stemness, epithelial–mesenchymal plasticity, drug-tolerant persister states, and niche-coupled metabolic adaptation. The assignment of an OnF state requires similarity to a well-defined fetal intestinal reference, attenuation of adult lineage identity, coordinated activation of fetal/regenerative programs, and orthogonal supporting evidence. In specific contexts, these programs may contribute to invasion, metastatic colonization, persistence, recurrence, and immune evasion; however, overlap between programs does not imply an identical biological identity or a shared causal mechanism.

Cellular plasticity enables tumor cells to transition among proliferative, differentiated, invasive, quiescent, and therapy-tolerant states, thereby adapting to hypoxia, inflammation, drug exposure, and evolving stromal niches (4–7). With advances in single-cell sequencing, spatial transcriptomics, and epigenetic technologies, oncofetal reprogramming (OnF) has been proposed as one route through which a subset of CRC cells acquires fetal-like or repair-like plasticity (**Figure 1**). Here, we define OnF as the coordinated reactivation of embryonic/fetal intestinal or injury-repair programs, rather than the upregulation of a single marker (8–10). This program may be accompanied by reduced lineage fidelity and an increased capacity for state transitions (11). However, the term OnF is sometimes applied too broadly to any YAP-high, stress-associated, EMP-like, or drug-tolerant state, thereby conflating developmental identity with generic outputs of plasticity.

We regard OnF as a testable route within the broader plasticity network of CRC, rather than as a common endpoint of all intestinal regeneration, EMP, stemness, DTP, immune exclusion, metastasis, or recurrence. The following sections sequentially examine its conceptual boundaries, operational criteria, physiological prototypes, regulatory networks, evidence hierarchy, relationships with major plasticity phenotypes, and relevance to clinical translation, while distinguishing direct evidence of OnF from overlapping plasticity mechanisms and indirect pathway associations (8, 9, 12, 13).

## 2 Definition and evidentiary boundaries of oncofetal reprogramming in CRC

### 2.1 Concepts and operational criteria

Early studies of oncofetal phenomena focused primarily on the re-expression of embryo-associated molecules, such as carcinoembryonic antigen (CEA) (14). Recent studies indicate that OnF reprogramming involves the remodeling of transcription factor networks and regulatory elements, enabling tumor cells to re-engage programs associated with embryonic development or tissue regeneration (8, 9).

Three related concepts should be distinguished. First, OnF reprogramming emphasizes the process through which developmental–regenerative programs are reactivated (8). Second, a fetal-like state (or OnF-like state) describes a cellular phenotype in which cancer cells resemble a well-defined fetal or regenerative reference at the transcriptional, chromatin, or functional level (10). Studies have shown that the normal intestinal epithelium can transiently enter a fetal-like regenerative state after injury, whereas some CRC models retain similar programs more persistently (15). Third, oncofetal plasticity describes the capacity of cells to transition between OnF-associated states and other states; its functional outputs may include lineage switching, restoration of stemness, EMP, or treatment tolerance, but none of these outputs is unique to OnF (11, 16).

Operationally, the identification of OnF should be based on multidimensional criteria. The core criteria include: robust mapping to a fetal intestinal reference for which the species, intestinal segment, and developmental stage are clearly defined; coordinated attenuation of adult intestinal epithelial lineage programs; and coordinated activation of a multigene fetal intestinal program rather than the upregulation of a single marker.

Supporting evidence includes: evidence from chromatin accessibility or enhancer activity; genetic or pharmacological perturbations that alter both the fetal program and functional phenotypes, with rescue evidence where appropriate; and lineage tracing, state reversibility, fetal-like organoid features, or spatial localization. Evidence of state reversibility can further strengthen the classification, although reversibility cannot always be measured directly in human samples (9, 10, 17, 18).

These criteria help avoid circular reasoning. A fetal state should not first be inferred from the activation of a particular pathway and then used to establish the OnF specificity of that same pathway; likewise, poor prognosis, recurrence, immune exclusion, or sensitivity to YAP inhibitors should not be retrospectively interpreted as evidence of OnF in the absence of fetal-reference similarity and evidence of changes in lineage state. **Table 1** summarizes the boundaries among OnF, dedifferentiation, EMP, and cancer stemness.

**Table 1:** Conceptual boundaries among OnF, dedifferentiation, EMP, and cancer stemness.

Concept	Core definition	Relationship to OnF	Minimum evidentiary requirement	Insufficient as a stand-alone criterion for OnF
Oncofetal reprogramming/OnF	Tumor cells reactivate a coordinated fetal intestinal/developmental program while attenuating adult intestinal epithelial lineage identity	Emphasizes the re-establishment of a fetal-like program	Mapping to a defined fetal reference, attenuation of adult lineage identity, and orthogonal validation	Expression of a single fetal-associated gene or marker
Dedifferentiation	Mature cells lose differentiated features and acquire a more primitive state	May loosen lineage constraints that facilitate OnF, but can also occur independently	Loss of mature identity accompanied by acquisition of progenitor-like function	Poor differentiation or reduced expression of lineage markers
Epithelial–mesenchymal plasticity	Reversible transitions between epithelial and mesenchymal-like states	May overlap with OnF but is not a required component	Mixed epithelial/mesenchymal features together with migratory or invasive function	EMT-marker expression or enhanced migratory capacity
Stemness/CSC phenotype	A functional property defined by self-renewal, clonogenicity, tumor initiation, and reconstruction of tumor hierarchy	OnF may provide a state scaffold for restoration of stemness in specific contexts but is neither necessary nor sufficient	Self-renewal and tumor-reconstitution assays	Expression of a single stemness marker

OnF should not be assigned on the basis of a single marker, pathway activity, histological poor differentiation, adverse prognosis, treatment tolerance, or spatial colocalization alone. Orthogonal evidence refers to support independent of the readout used to define the state, including chromatin/enhancer analyses; genetic or pharmacological perturbation with rescue; lineage tracing; state reversibility; functional organoid phenotypes; or spatially resolved analyses that simultaneously resolve tumor epithelial states.

### 2.2 Evidence hierarchy for OnF states

To prevent conceptual overextension, the evidence can be classified into three categories. Direct evidence of OnF requires the same biological system to demonstrate both clear similarity to a defined fetal reference and internally coherent reactivation of a fetal intestinal program, together with at least one form of orthogonal support. Second, overlapping plasticity mechanisms are those that can coexist with OnF, promote its establishment or maintenance, or be selected in OnF states, but can also operate independently in the absence of a complete fetal reference and orthogonal validation; these include subsets of EMP, dynamic stemness, DTP, dedifferentiation, and related signaling pathways. Third, indirect associative evidence mainly includes expression correlations, associations with clinical outcomes, niche enrichment, and spatial colocalization. Such evidence may suggest that OnF-like cells are present in a particular environment, but it cannot directly demonstrate the reactivation of a fetal program or establish the direction of causality.

Importantly, the level of evidence is not a fixed property of a particular molecule or pathway but depends on the reference framework, state readouts, experimental perturbations, and functional endpoints used in a given study. The same pathway may constitute direct evidence, an overlapping mechanism, or an indirect association in different studies. For example, YAP/TAZ, AP-1, or TGF-β can be classified as a direct OnF mechanism only when the criteria for fetal-reference similarity, altered lineage identity, and orthogonal validation are all met; if a study demonstrates only pathway activation, EMP, drug resistance, or spatial enrichment, the findings should instead be classified as an overlapping mechanism or an indirect association, as appropriate. Details are provided in **Table 2**.

**Table 2:** Evidence hierarchy for OnF in CRC.

Evidence category	Representative findings	Level of support	Biological implication	Notes	Contextual support
Direct evidence of CRC OnF	Similarity to a defined fetal reference, attenuation of adult lineage identity, coordinated activation of a fetal program, and orthogonal functional or chromatin support	Strong evidence	Supports the existence of OnF states in CRC	At least one form of orthogonal evidence is required	(6, 9, 10, 29–31)
Overlapping plasticity mechanisms	EMP, CSC and DTP states, as well as YAP/TAZ-, AP-1-, or TGF-β-associated programs lacking fetal-reference validation	Mechanistic support	Explains state transitions, invasion, and treatment resistance	May coexist with OnF or arise independently	(7, 12, 15, 24, 27, 28, 52, 62)
Indirect niche associations	CAF/ECM remodeling, high TGF-β activity, inflammation/immune exclusion, SPP1-positive macrophages, and spatial colocalization	Indirect evidence	May support or select for OnF-like states	Co-occurrence or spatial localization does not establish causality	(41, 64, 66, 107–109)

Orthogonal support should be independent of the same expression readout used to define the state. Spatial localization without concurrent resolution of fetal-reference similarity in tumor epithelial cells constitutes indirect evidence only. In individual studies that include fetal-reference and perturbational evidence, YAP/TAZ, AP-1, or TGF-β may be upgraded to direct mechanistic support; evidence level should not be predetermined by pathway name.

Causal terminology should be standardized according to the level of evidence. When a study includes genetic or pharmacological perturbations and demonstrates concomitant changes in the fetal program and functional endpoints, particularly when supported by rescue experiments, the terms “drive”, “maintain”, or “mediate” are appropriate; when the evidence is limited to co-occurrence, spatial localization, or expression associations, the terms “associate with”, “accompany”, or “be enriched in” should be used; when the evidence is derived mainly from other cancer types or general repair models, the conclusion should be limited to “may be involved” or “provides mechanistic plausibility”.

### 2.3 Fetal-like regeneration and alternative intestinal injury-repair routes

The adult intestine contains multiple context-dependent regenerative routes. Under homeostatic conditions, the normal intestinal epithelium relies on LGR5-positive crypt base columnar stem cells to sustain tissue renewal (19). Following irradiation, inflammation, chemical injury, or depletion of LGR5-positive cells, repair can involve surviving LGR5-positive cells, the traditionally designated +4/reserve cells, facultative progenitors, secretory-lineage progenitors, dedifferentiated mature epithelial cells, and revival stem cell populations (12, 15, 20–22). These repair routes are neither conceptually nor functionally equivalent, and their relative contributions depend on the type of injury, intestinal segment, time point, and lineage-tracing tools used.

Some of these repair responses exhibit fetal-like features, including attenuation of adult differentiation programs, YAP/TAZ activation, ECM remodeling, and transcriptional similarity to the developing intestine (12, 13, 23–26). Ayyaz et al. found that injury-induced revival stem cells can contribute to the reconstitution of the LGR5-positive compartment (27). Through matrix and pathway perturbations, Yui et al. linked the ECM–FAK/Src–YAP/TAZ axis to a primitive fetal-like colonic state (15). These studies provide a physiological template for understanding how tumors may stabilize repair states that are normally transient, but their findings cannot be directly extrapolated as a universal mechanism in CRC.

Inflammation, cytokines, and myeloid- or stromal-derived signals can promote epithelial survival, dedifferentiation, stem cell-like behavior, and therapeutic adaptation, but these changes are not automatically equivalent to OnF reprogramming. Such changes should be classified as OnF-related reprogramming only when inflammatory exposure concurrently induces clear similarity to a fetal intestinal reference, attenuation of adult lineage identity, and coordinated activation of a fetal program, with supporting evidence from chromatin analyses or functional perturbations (8, 9, 28, 29). In other settings, inflammatory signaling is more appropriately described as a plasticity mechanism that overlaps with OnF or as a factor that may select for and maintain pre-existing OnF-like cells.

Under the influence of oncogenic mutations, sustained mechanical stress, matrix remodeling, and treatment exposure, some CRCs may retain for prolonged periods fetal-like repair programs that are normally transient (10, 23, 30, 31). This “pathological fixation” model has strong explanatory value only when supported by longitudinal or perturbational evidence and should not be interpreted as indicating that a single repair trajectory can account for all tumors and all disease stages. The relative contribution of fetal-like repair is likely to differ among early adenomas, invasive fronts, metastatic lesions, and post-treatment residual disease.

## 3 Regulatory networks and their context dependence

**Figure 2:**
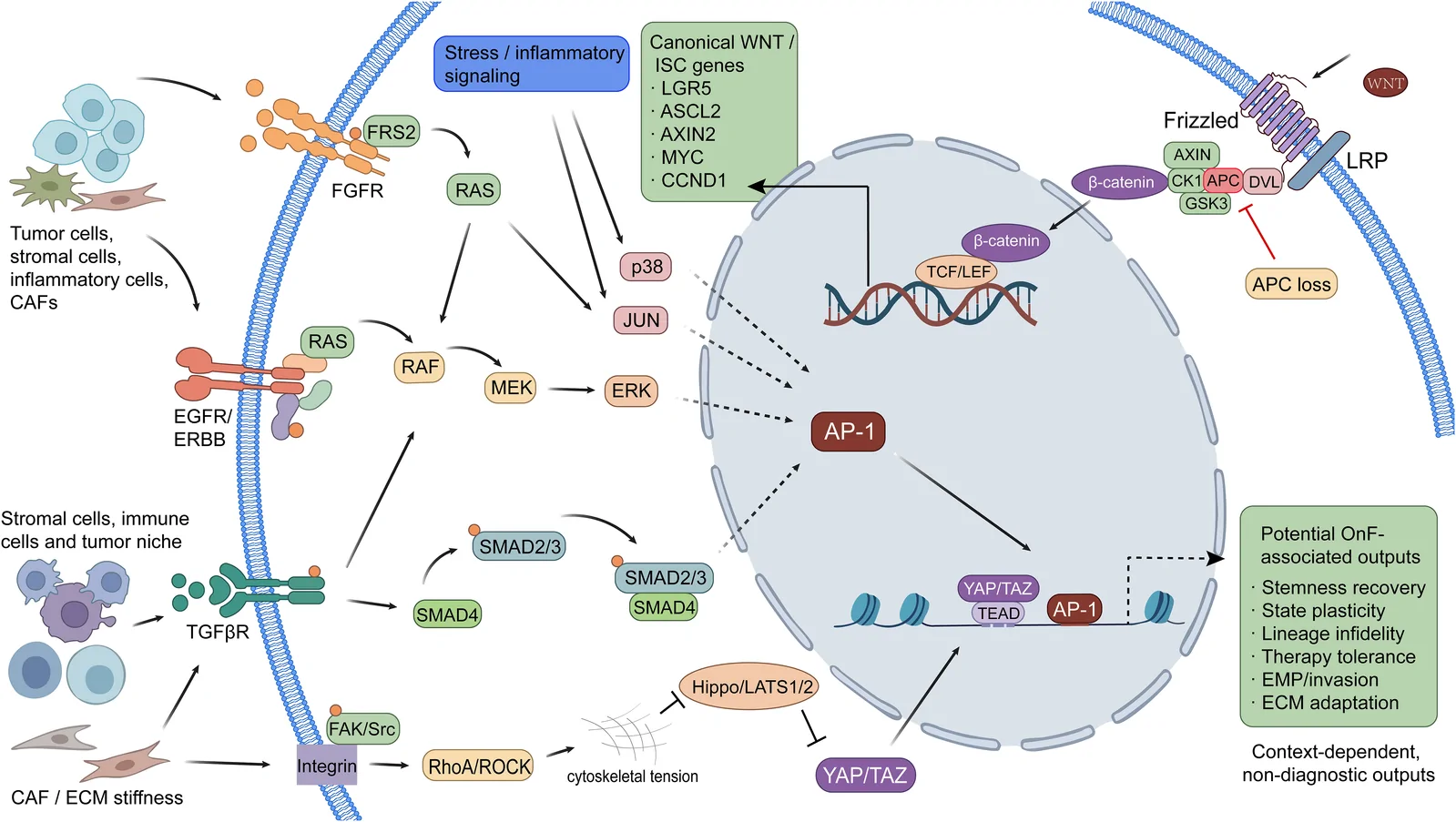
Context-dependent regulatory network associated with oncofetal reprogramming in colorectal cancer. APC loss and canonical Wnt signaling primarily constitute a genotype-dependent background and are insufficient on their own to establish an OnF identity. EGFR/FGFR–RAS–MAPK signaling can enhance AP-1 activity. ECM stiffening and integrin–FAK/Src–RhoA/ROCK signaling can promote YAP/TAZ–TEAD activation by relieving Hippo/LATS-mediated inhibition of YAP/TAZ. In most CRC studies, TGF-β/SMAD, inflammatory, and stroma-derived signals are primarily implicated in EMP, immune exclusion, stromal remodeling, or the establishment of locally permissive niches. In a subset of CRC models that also show similarity to fetal intestinal references, attenuation of adult lineage identity, and evidence from functional perturbation, the coordinated activity of YAP/TAZ–TEAD and AP-1 contributes to the establishment or maintenance of fetal/regenerative transcriptional programs. Solid lines indicate relatively direct pathway or functional support, whereas dashed lines indicate context-dependent inputs, putative regulation, or associations. The green boxes on the right indicate context-dependent outputs that may accompany OnF, including restoration of stemness, state plasticity, lineage infidelity, treatment tolerance, EMP/invasion, and ECM adaptation. These outputs are not defining components of OnF and can also arise independently through non-OnF mechanisms. No single pathway, output, or marker is sufficient to define OnF. Its assignment still requires similarity to a fetal intestinal reference, attenuation of adult lineage identity, coordinated activation of fetal/regenerative programs, and orthogonal functional or chromatin evidence.

OnF reprogramming in CRC is shaped by context-dependent interactions among oncogenic background, transcriptional effectors, growth-factor signaling, and microenvironmental inputs. Wnt/APC alterations primarily provide a genetic and lineage context, whereas YAP/TAZ–TEAD and AP-1 act as major transcriptional effectors in selected models with direct OnF evidence. EGFR/FGFR, TGF-β, and ECM mechanotransduction mainly function as regulatory inputs (**Figure 2**).

### 3.1 Wnt/APC aberrations provide a permissive context

The role of Wnt signaling in cancer has been extensively characterized, with its principal functions including the promotion of tumor proliferation, metastasis, stem-cell maintenance, and drug resistance (32, 33). APC alterations are present in approximately 70%–80% of cases across different cohorts, whereas Wnt pathway aberrations are detectable in an even larger proportion of tumors (19, 34, 35). This background supports adenoma initiation and canonical LGR5/ASCL2-associated stemness (36, 37), but Wnt activation alone does not establish a fetal identity. Recent single-cell and organoid studies suggest that, during progression, metastasis, or treatment, a subset of CRC cells can transition from a crypt-like Wnt-high state to a lineage-disordered or fetal progenitor-like state (6, 9, 38). Genotype can modify this transition: APC-mutant tumors are generally less dependent on exogenous Wnt ligands, whereas tumors harboring RNF43 mutations or RSPO fusions may retain ligand dependence and respond differently to upstream interventions such as PORCN inhibition (23, 39–45).

Anatomical location and molecular subtype further modify Wnt output and its interactions with other pathways. Left- and right-sided CRCs differ in their mutational profiles, immune contexture, stromal composition, and treatment responses; MSI status, CMS/CRIS subtype, primary tumor location, and metastatic organ also shape the immune and stromal context. Wnt-high and YAP-high states should not be generalized as a universal dichotomy, because cells may occupy mixed or transitional states. The relative contributions of different pathways may also vary among primary tumors, invasive fronts, residual lesions, and metastatic sites (6, 16, 17, 28, 30).

### 3.2 YAP/TAZ–TEAD and AP-1: strong mechanistic evidence but context-dependent effects

YAP/TAZ are transcriptional coactivators that are inhibited by the Hippo kinase cascade. MST1/2 activate LATS1/2, which phosphorylate YAP/TAZ and restrict their nuclear activity. By contrast, loss of polarity, mechanical tension, integrin–FAK/Src–RhoA/ROCK signaling, and inputs from certain growth factors can attenuate this inhibition, promote nuclear activation of YAP/TAZ–TEAD, and regulate the expression of genes involved in proliferation, survival, migration, matrix adaptation, and regeneration (46–50). In CRC models with program-level evidence of similarity to fetal references, the OnF state is accompanied by increased TEAD and AP-1 motif activity and enhanced accessibility of associated enhancers. Genetic and pharmacological perturbations further support a functional contribution of the YAP/TAZ–TEAD/AP-1 module to state transitions (6, 9).

The effects of YAP/TAZ are not fixed. During normal repair, transient activation can expand regenerative compartments (15, 23); in some APC-driven or LGR5-positive models, strong YAP activity suppresses canonical Wnt output and proliferation, and regenerative reprogramming may even constrain primary tumor expansion (23, 51). However, in the contexts of invasion, metastasis, or treatment exposure, low-Wnt, mechanically adapted, or regenerative states may be associated with greater survival, migratory capacity, and relapse potential (7, 9, 52). YAP/TAZ therefore cannot be uniformly characterized as either oncogenic or tumor suppressive; their effects depend on the intensity and duration of activation, subcellular localization, genotype, tissue compartment, and treatment history.

AP-1 comprises members of the JUN, FOS, FOSL1, FOSL2, and ATF families and responds to MAPK, inflammatory, stress, and mechanical signals (53, 54). At the enhancer level, YAP/TEAD and AP-1 can cooperatively occupy regulatory regions and alter chromatin accessibility (49, 50, 53, 55). In CRC models providing direct evidence of OnF, perturbation of either YAP or AP-1 affects both the fetal program and treatment tolerance, supporting their roles in mediating specific OnF state transitions (9). In systems lacking fetal-reference or perturbational evidence, AP-1 activation is more appropriately described as being associated with stress-related and regenerative-like programs.

Individual molecules should be used as within-study readouts rather than as standalone diagnostic markers. Functional perturbation experiments have implicated L1CAM in matrix adhesion, anoikis resistance, and metastatic initiation in CRC cells (52, 56); EMP1 may mark or contribute to cellular states associated with post-treatment residual disease, regenerative-like features, and postoperative recurrence (7); CYR61/CCN1 and CTGF/CCN2 are common YAP/TEAD outputs that can link YAP nuclear translocation to ECM remodeling and mechanical feedback (49). These molecules may overlap with OnF/EMP, but their upregulation alone is insufficient to define OnF.

### 3.3 Extrinsic regulatory inputs and microenvironmental niches

#### 3.3.1 EGFR/FGFR–MAPK growth-factor inputs

EGF/EGFR and FGF/FGFR are important growth-factor inputs in intestinal injury repair and therapeutic adaptation in CRC. Although mediated by distinct receptor–ligand systems, both can converge on the RAS–RAF–MEK–ERK and JNK/p38 pathways and thereby influence AP-1-mediated stress and regenerative transcriptional programs (54, 57–59). Current evidence supports their roles in regulating cell states; however, they should be described as mediating OnF transitions only when both fetal-reference and orthogonal functional criteria are satisfied.

During normal intestinal injury repair, EGFR ligands are commonly released by damaged epithelial cells, inflammatory cells, and stromal cells and contribute to epithelial survival, migration, proliferation, and restoration of the mucosal barrier (60). In the context of KRAS or BRAF mutations, MAPK signaling can remain constitutively active and become partly uncoupled from upstream EGFR regulation (59, 61, 62). FGF/FGFR signaling has more pronounced developmental and stromal-niche features (10, 57). It can be influenced by CAFs and the local matrix and is associated with CRC cell proliferation, survival, and adaptation to hypoxia, inflammation, matrix remodeling, and therapeutic pressure (10, 58, 63). If these pathways induce the program-level OnF criteria described above, they may promote OnF transitions; however, sustained MAPK activation can also generate proliferative or stress-tolerant states that lack fetal features.

#### 3.3.2 The TGF-β–CAF–ECM mechanical niche

TGF-β exhibits pronounced stage-dependent effects in CRC. In normal or early-stage epithelium, TGF-β can restrict proliferation, whereas after tumor cells escape its growth-inhibitory effects, it can promote EMP, matrix remodeling, immune exclusion, and the formation of metastatic niches (64–71). TGF-β derived from CAFs and other stromal sources can promote CAF activation, collagen deposition, and matrix stiffening, thereby collectively shaping a niche that favors YAP/TAZ-dependent mechanical adaptation and the maintenance of cellular plasticity (31, 41, 67, 68, 72, 73). In general models of intestinal regeneration, TGFB1 stimulation and pathway activation can induce fetal-like reprogramming (69), providing mechanistic plausibility for its involvement in OnF. In most CRC studies, the evidence for TGF-β primarily supports niche remodeling or EMP rather than a direct fetal identity.

ECM stiffening and integrin engagement can activate FAK/Src and RhoA/ROCK, increase actomyosin tension, and attenuate Hippo/LATS-mediated inhibition of YAP/TAZ, thereby promoting the nuclear translocation of YAP/TAZ (15, 31, 67, 72, 74). YAP/TEAD can subsequently upregulate matrix-remodeling genes such as CTGF, CYR61, and FN1, forming a feedback loop (15, 31, 49, 73). Recent CRC organoid studies using mechanical and receptor perturbations have shown that integrins and mechanosensitive calcium channels can jointly mediate the formation of fetal-like states, providing relatively direct evidence that mechanical inputs participate in OnF reprogramming (31). This mechanical adaptation axis may partly explain why some OnF-like states are enriched at invasive fronts and associated with metastatic dissemination. In these regions, tumor cells must adapt to matrix resistance, tensile forces, and changes in tissue stiffness, and YAP/TAZ activation can support their adhesion, survival, and migratory adaptation (15, 31, 75, 76).

#### 3.3.3 Crosstalk between inflammation-related signaling and OnF

NF-κB is an important regulatory node linking inflammatory stimuli to cell-state transitions in CRC. In genetic models, NF-κB activation can cooperate with Wnt aberrations to promote the dedifferentiation of mature intestinal epithelial cells and confer tumor-initiating capacity (28). This study provides functional evidence of inflammation-driven cellular plasticity; however, because it lacks a clearly defined fetal intestinal reference, it is more appropriately regarded as a dedifferentiation mechanism that intersects with OnF rather than as direct evidence of OnF.

The JAK–STAT pathway primarily regulates CRC cell survival, stemness, and therapeutic adaptation through cytokine signaling. IL-6/gp130–STAT3 signaling can promote inflammation-associated tumorigenesis and epithelial-cell survival; the IL-22–STAT3 axis can enhance stemness-associated features in CRC cells; and tumor-associated macrophage-derived IL-6 can promote STAT3-dependent migration and EMP phenotypes (77–79). These studies support the involvement of JAK–STAT signaling in maintaining inflammation-induced plasticity, but current evidence remains insufficient to establish that it broadly mediates the reactivation of fetal programs.

EGFR/FGFR, TGF-β, CAF–ECM signaling, and inflammation-related signals should therefore be regarded as regulatory inputs that may intersect with OnF. Their evidentiary classification depends on whether a given study concurrently demonstrates activation of a fetal program, loss of adult lineage identity, and orthogonal functional support, rather than solely on pathway activation or shared malignant phenotypes.

### 3.4 Notch signaling: progenitor maintenance, differentiation blockade, and CRC plasticity

Notch signaling maintains proliferative crypt progenitors and suppresses secretory-lineage differentiation; γ-secretase inhibition redirects proliferating cells in normal crypts and adenomas toward goblet-cell differentiation (80, 81). In genetically engineered CRC models, epithelial Notch activation can remodel the tumor microenvironment and mediate phenotypes associated with poor-prognosis subtypes and metastasis. Pathway inhibition or genetic perturbation can attenuate these functional endpoints (82). These findings support a role for Notch in maintaining specific progenitor-like or plastic cell states, but the relevant studies have not consistently used clearly defined fetal intestinal references; Notch should therefore not be directly described as driving OnF.

CDX2 can serve as a reference axis for lineage fidelity. Loss of Cdx2 can induce homeotic and gastric-like lineage transitions *in vivo (*83, 84). In human CRC cohorts, reduced CDX2 expression is associated with poor differentiation, specific molecular features, and worse clinical outcomes (85). These findings suggest that CDX2 loss can permit lineage infidelity, but they remain insufficient to establish OnF; a coherent fetal intestinal program, rather than another form of lineage conversion, must also be demonstrated.

### 3.5 Chromatin remodeling and the evidentiary boundaries of “state memory”

OnF-associated states may be accompanied by increased accessibility of TEAD/AP-1 motifs, enhancer activation, and aberrant chromatin landscapes (9, 55, 86). In CRC models providing direct evidence of OnF, the coupling of TEAD/AP-1 motif activity to fetal-like programs, together with perturbational findings, primarily supports a role for epigenetic regulation in state transitions (9). Other epigenomic studies in CRC have identified YAP/TAZ-associated enhancer landscapes and molecular subtype-associated changes in chromatin accessibility (49, 86). However, whether these alterations persist and what functional significance they have remain incompletely understood. The term “chromatin-state memory” should be reserved for experimentally validated phenomena in which a state persists after withdrawal of the inducing signal and leads to more rapid re-entry into that state or to altered cell-fate behavior.

Current evidence is insufficient to assume that all OnF states involve stable epimutations or persistent three-dimensional chromatin reorganization. Single-cell ATAC-seq data are sparse, motif activity is inferred rather than directly measured, and bulk epigenomic profiles are also susceptible to stromal admixture. Orthogonal perturbations and longitudinal sampling are required to distinguish causal enhancer remodeling from transient consequences of state transitions (17, 18, 86).

## 4 Relationship of OnF to major plasticity phenotypes in CRC

### 4.1 EMP and regenerative-like migratory states

Epithelial–mesenchymal plasticity (EMP) is a major manifestation of cell-state transitions in colorectal cancer. Rather than undergoing the unidirectional conversion from an epithelial to a mesenchymal phenotype implied by the traditional view of EMT, CRC cells often retain epithelial features and occupy partial EMT or hybrid E/M states (87–90). While retaining aspects of epithelial adhesion and collective behavior, these cells can acquire motility, matrix adaptability, and the capacity for collective migration (89, 91–94). When a defined fetal-like transcriptional signature co-occurs with programs associated with YAP/TAZ, AP-1, L1CAM, or EMP1, OnF may functionally overlap with certain regenerative-like migratory states (7, 12, 15, 27, 49, 52, 95).

OnF should not be regarded as the origin of all EMP states. TGF-β, hypoxia, inflammation, oncogenic mutations, and metabolic stress can all induce partial EMT in the absence of a fetal program (28, 41, 96, 97). An EMP-like cell state should be classified as an OnF-associated regenerative-like migratory state only when it also exhibits similarity to fetal reference states, attenuation of adult lineage programs, and corresponding functional or chromatin evidence. Elevated VIM, ZEB1, or MMPs supports the presence of EMP but does not, by itself, establish OnF.

Such overlapping states may be particularly relevant during metastasis. Some CRC cells at invasive fronts and in residual lesions after therapy can concurrently display fetal-like, stress-like, and partial EMT features, which have been associated with adaptation, departure from the original niche, and re-establishment of growth at distant sites (2, 7, 9, 30, 52). However, this relationship remains model- and context-dependent, and CRC dissemination can also be supported by non-OnF EMP, inflammatory, or metabolic adaptation programs.

### 4.2 Canonical and alternative stemness

LGR5-positive CRC cells sustain primary tumor growth, and depletion of this population can transiently restrict tumor expansion (40, 98). Lineage-tracing and metastasis models indicate that some LGR5-negative cells can reacquire stemness after LGR5-positive cells are lost; disseminated LGR5-negative cells can also regenerate LGR5-positive cells after colonizing distant sites and re-establish the tumor hierarchy within metastatic lesions (40, 95).

OnF provides one explanation for the reversible restoration of stemness in CRC cells. Fetal-like or regenerative-like programs can relax the lineage constraints imposed by the adult intestinal epithelium, weaken mature intestinal epithelial identity, and enhance lineage plasticity, thereby enabling dynamic state transitions among migration, colonization, and regenerative expansion (6, 10, 99, 100). Rather than replacing the canonical LGR5-positive cancer stem cell model, OnF extends it: CRC stemness may be maintained by a relatively stable LGR5-positive adult intestinal stem cell-like population or re-established through fetal-like or regenerative-like reprogramming under microenvironmental stress or during metastasis.

Alternative stemness can also arise through NF-κB, JAK–STAT, or Notch signaling, metabolic remodeling, slow-cycling states, or support from local niches (62, 96, 97, 101). Functional stemness should be demonstrated by self-renewal, tumor initiation, and lineage reconstitution rather than inferred from a single marker. OnF is neither necessary nor sufficient for all CRC stemness, but in specific genotypic and microenvironmental contexts, it may provide a state scaffold that favors stemness restoration.

### 4.3 Induction by therapeutic pressure, DTP states, and recurrence

Chemotherapy, radiotherapy, targeted therapy, and insufficient nutrient and oxygen supply can induce tumor cells to enter reversible survival states characterized by low proliferation, apoptosis resistance, and metabolic remodeling (24, 102–104). DTP cells typically exhibit slowed proliferation, reduced drug sensitivity, increased apoptosis resistance, and enhanced metabolic adaptation, and can resume proliferation after treatment withdrawal (62, 105, 106). DTP states generated by different treatments and models do not necessarily share the same developmental identity.

The link between OnF and DTP states derives mainly from the diapause-like or fetal progenitor-like features observed in some CRC persister cells. In patient-derived CRC models, Rehman et al. found that chemotherapy induced a low-proliferative state resembling embryonic diapause; barcode analysis suggested that multiple cancer cell populations can enter this state, rather than the state arising solely through expansion of a small number of pre-existing resistant clones (24). This finding supports the view that treatment tolerance can arise through widespread state transitions; however, although diapause-like programs are conceptually related to OnF, the two are not equivalent.

After chemotherapy, MEX3A-positive CRC persister cells can adopt a transient state resembling YAP-positive fetal intestinal progenitors (103). In addition, CLU-positive revival stem cells can be induced during intestinal injury repair, and patient-derived CRC organoids can upregulate revival stem cell-associated markers after chemotherapy (27, 104). These findings suggest that persister states with fetal-like or regenerative-like features may contribute to DTP formation and post-treatment regeneration; however, injury-response markers such as CLU are not sufficient on their own to define an OnF state.

In direct OnF models, a YAP/AP-1-associated fetal program is mechanistically linked to FOLFIRI tolerance, and disrupting the OnF program in combination with standard therapy can suppress tumor regeneration in these models (9). These findings support the possibility that OnF provides a reversible state scaffold for a subset of DTPs, but they do not justify attributing all DTP states or recurrence to OnF. The most compelling evidence linking OnF to recurrence should demonstrate both the formation of a fetal state and, through genetic or pharmacological perturbation, its functional contribution to persister-cell survival and regrowth.

### 4.4 Immune and stromal niches

The formation and maintenance of OnF-like states depend not only on tumor cell-intrinsic genotypes but also on the local stromal and immune environment (30, 107–109). Spatial studies have shown that invasive fronts, CAF-enriched regions, hypoxic regions, and post-treatment residual areas harbor distinct cell states (66, 108, 110). Some OnF-like or EMP-like cells are enriched at invasive fronts and in stroma-rich regions, whereas LGR5/ASCL2/Wnt-high states are more frequently found in crypt-like and proliferative regions (30, 107–109, 111); by themselves, these spatial observations primarily support co-occurrence and localization.

An early CRC spatial-omics study found that OnF-like epithelial cells were spatially adjacent to specific trophocyte-like CAFs; co-culture experiments further showed that these CAFs can induce tumor epithelial cells to transition into an OnF state, providing functional evidence for stromal niche-induced reprogramming (30). In another genetically engineered study, SOX17-driven fetal-like CRC cells downregulated MHC-I-associated antigen-presentation programs and reduced recognition by cytotoxic T cells, providing a relatively direct mechanistic link between fetal-like identity and immune evasion (29).

By contrast, high TGF-β activity, collagen deposition, enrichment of SPP1-positive macrophages, and exclusion of CD8-positive T cells are common features of immunosuppressive CRC niches but are not specific to OnF states (41, 64–66, 107–109). These factors may create an environment favorable to the survival and selection of OnF/EMP-like cells, but they can also support immune evasion through independent mechanisms. The two processes may reinforce each other bidirectionally; however, the direction of causality must be established through time-resolved perturbation experiments and paired spatial sampling and cannot be inferred solely from expression correlations or spatial colocalization (29, 41, 66, 107, 112).

### 4.5 Molecular subtype, tumor site, disease stage, and treatment context

The prevalence and functional role of OnF states are unlikely to be uniform across all CRCs. APC-mutant, RNF43/RSPO ligand-dependent, RAS-mutant, BRAF-mutant, MSI-high, and MSS tumors are subject to distinct signaling and immune constraints. Stroma-rich CMS4 tumors may receive stronger TGF-β/CAF/ECM inputs, whereas epithelial, Wnt-dominant CMS2 tumors arise in a distinct context; MSI-high tumors are additionally shaped by unique interferon signaling and immune selection pressures. Right- and left-sided primary tumors differ in genotype, epigenetic history, microbiota, and treatment sensitivity; primary tumors, liver metastases, peritoneal metastases, and residual lesions also occupy distinct biomechanical and immune niches (16, 17, 64, 96, 100, 107–109).

Future studies evaluating OnF should report tumor site, stage, prior treatment, sampling site, molecular subtype, and key genotypes. Conclusions derived from a single organoid model, a single genetically engineered mouse model, or a single metastatic site should not be generalized to all CRCs without cross-model validation (6, 10, 17, 30, 96, 113).

## 5 Therapeutic intervention and clinical translation

### 5.1 Therapeutic strategies stratified by stage of evidence

The therapeutic significance of OnF reprogramming does not lie in providing a stand-alone target that could replace existing CRC treatments. Rather, it suggests that therapeutic strategies should address not only the elimination of tumor cells but also the capacity of residual cells to undergo state transitions and re-establish tumors under therapeutic pressure (9, 13, 24, 114). However, no drug has yet been approved specifically for patients with OnF-high CRC as defined by the operational criteria used in this review. Standard chemotherapy, anti-EGFR therapy, BRAF-targeted therapy, immunotherapy for MSI-high disease, and surgery should remain standard-of-care treatments according to the clinical context (3, 115). Unless a clinical study enrolls patients using validated OnF criteria, the intervention should be described as targeting an OnF-associated axis rather than as an established anti-OnF therapy. For clarity, we divide the relevant strategies into four categories according to the directness of their relationship to the OnF state and their degree of clinical maturity. This classification is intended solely to organize the discussion and does not constitute a validated hierarchy of clinical evidence. Category 1 comprises approved standard-of-care CRC regimens that may affect plasticity but are not anti-OnF therapies. Category 2 comprises clinical-stage agents targeting OnF-associated regulatory axes, including PORCN, TEAD, TGF-β, FAK, and MAPK. Category 3 comprises preclinical strategies, including YAP/TEAD degraders, approaches that reduce YAP stability through AURKA or CDK4/6 inhibition, differentiation therapy, and selected epigenetic interventions. Category 4 comprises conceptual strategies, such as intermittent dosing triggered by an increase in OnF, maintenance therapy aimed at suppressing plasticity, or the addition of an OnF-axis inhibitor when ctDNA becomes positive. **Table 3** lists only registered clinical studies and programs involving dynamic ctDNA-based stratification. Preclinical or conceptual strategies, including YAP/TEAD degradation, AURKA-targeted intervention, differentiation or epigenetic therapy, and intermittent dosing, are discussed in the main text but are not all included in **Table 3**.

**Table 3:** Representative clinical and translational studies targeting pathways associated with OnF-related plasticity.

Translational axis/strategy	Intervention	Registration number	Phase/design	Study population	OnF relevance	Status	Key evidence	Key limitations	Clinical or translational publication
Ligand-dependent Wnt	Zamaporvint (RXC004) ± nivolumab	NCT04907539	Phase II; open-label, multi-arm	MSS mCRC with RNF43 mutations or RSPO fusions	Blocks upstream Wnt dependence	Completed	Limited single-agent activity; preliminary signals with combination immunotherapy	Small sample size; no randomized control; no OnF-based stratification	(117)
Ligand-dependent Wnt	ETC-1922159 (ETC-159) ± pembrolizumab	NCT02521844	Phase Ia/b; dose escalation/expansion	Advanced solid tumors	Inhibits PORCN and Wnt secretion	Active, not recruiting	Established a safe dose and provided preliminary pharmacodynamic evidence	Basket solid-tumor study; limited CRC data	–
Combined Wnt/BRAF/EGFR inhibition	WNT974 + encorafenib + cetuximab	NCT02278133	Phase Ib/II; open-label, dose escalation	BRAF V600E, KRAS-wild-type mCRC	Combined inhibition of Wnt and MAPK/EGFR	Terminated	Demonstrated feasibility of the combination, but with substantial toxicity	Limited enrollment; bone toxicity; lack of efficacy validation	(140)
Direct TEAD inhibition	VT3989	NCT04665206	Phase I/II; dose escalation/expansion	Solid tumors with Hippo pathway alterations, predominantly mesothelioma	Direct inhibition of TEAD-mediated transcription	Recruiting	Generally well tolerated; preliminary antitumor signals reported, mainly in mesothelioma cohorts	Lack of CRC data and OnF-based stratification	(119)
Direct TEAD inhibition	SW-682	NCT06251310	Phase I; dose escalation/expansion	Advanced solid tumors and tumors with Hippo pathway alterations	Targets TEAD-dependent transcription	Recruiting	Currently in safety and dose-finding evaluation	No mature efficacy data; no dedicated CRC cohort	–
YAP/TAZ–TEAD inhibition	IAG933	NCT04857372	Phase I; dose escalation/expansion	Tumors with NF2/LATS alterations or YAP/TAZ fusions	Disrupts the YAP/TAZ–TEAD interaction	Active, not recruiting	Primary dose-escalation evaluation completed	No CRC-specific results; no OnF-based enrollment	(118)
TGF-β/immune niche	Vactosertib + pembrolizumab	NCT03724851	Phase Ib/IIa; open-label, multicenter	Previously treated mCRC and gastric cancer	Inhibits TGF-β-associated immune exclusion	Completed	Manageable safety and preliminary clinical activity signals in a nonrandomized setting	Nonrandomized; CMS4 is not equivalent to OnF	(141)
MAPK/FAK/EGFR plasticity	Avutometinib + defactinib + cetuximab	NCT06369259	Phase II; open-label, single-arm	Advanced CRC resistant to anti-EGFR therapy	Inhibits MAPK reactivation and FAK-mediated adaptation	Recruiting	No formal results available	Single-arm study; OnF signature not assessed	–
Indirect modulation via CDK4/6	Abemaciclib + 5-FU	NCT06654037	Phase I; dose exploration	Refractory mCRC	May indirectly reduce YAP activity	Recruiting	Primary evaluation of safety, dose, and pharmacodynamic endpoints	OnF relevance is based mainly on preclinical evidence	–
ctDNA-guided adjuvant therapy	CIRCULATE-US/NRG-GI008	NCT05174169	Phase II/III; randomized, ctDNA-stratified	R0-resected stage II–III colon cancer	Used for MRD and recurrence-risk stratification	Recruiting	Compares ctDNA-guided treatment escalation or de-escalation	ctDNA does not directly measure the OnF state	(142)
Intervention for ctDNA-positive MRD	FTD/TPI versus placebo (ALTAIR)	NCT04457297	Phase III; randomized, double-blind	Postoperative ctDNA-positive CRC	Targets molecular recurrence rather than OnF directly	Completed	DFS improved, but not significantly; primary endpoint not met.	Hematologic toxicity; no OnF biomarker	(143)

Clinical trial registration information was accessed on July 20, 2026. “OnF relevance” in this table primarily denotes mechanistic overlap or overlap with plasticity pathways and does not indicate established direct anti-OnF efficacy. ctDNA primarily reflects molecular residual disease and recurrence risk and cannot directly characterize the OnF state.

### 5.2 Targeting Wnt dependence, YAP/TEAD, and related pathways

In most CRCs, Wnt alterations occur downstream in the pathway, most commonly through APC inactivation. PORCN inhibition is therefore more biologically plausible in ligand-dependent CRCs harboring RNF43 mutations or RSPO fusions than in most APC-mutant tumors (44, 116, 117). Inhibition of canonical Wnt-high stemness may also fail to prevent cells from transitioning into Wnt-low, YAP/AP-1-high, or other tolerant states. Wnt inhibition should therefore be regarded as a therapeutic strategy for selected molecular subtypes rather than as a broadly applicable OnF-associated intervention.

TEAD palmitoylation and the YAP/TAZ–TEAD protein interaction are currently major areas of drug development (118, 119). The oral TEAD palmitoylation inhibitor VT3989 has been evaluated in a phase I/II of refractory solid tumors, with enrollment primarily comprising patients with Hippo pathway-altered tumors such as mesothelioma (119). IAG933 and other inhibitors of the YAP–TEAD interface have shown antitumor activity in models with Hippo pathway abnormalities or RAS–MAPK alterations and in CRC cells (118, 120). However, existing studies have generally not selected patients according to an OnF signature or included suppression of fetal-like programs as a pharmacodynamic endpoint. Their clinical value in OnF-high CRC therefore remains uncertain. YAP/TAZ also contribute to normal intestinal homeostasis and injury repair, and the therapeutic window must therefore be evaluated carefully (118–120).

Reducing YAP/TAZ stability represents another potential intervention strategy. AURKA can maintain YAP1/TAZ activity through a Hippo-independent mechanism and contributes to primary or acquired resistance to anti-EGFR therapy in CRC. In CRC models, AURKA inhibition reduces YAP1/TAZ stability and enhances the therapeutic effects of cetuximab (121, 122). CDK4/6 inhibition can promote YAP1 degradation through a DUB3-associated mechanism and restrict CRC progression (123).

PROTAC and bioPROTAC technologies can also directly reduce the abundance of YAP or TEAD proteins (124–126). In principle, these strategies may reduce compensation among TEAD paralogs and overcome incomplete target inhibition. However, evidence in CRC remains largely limited to cellular and animal models. Delivery efficiency, tissue selectivity, duration of degradation, immunogenicity, and toxicity to normal tissues all require further validation. AURKA inhibition, CDK4/6 inhibition, and protein-degradation strategies also affect mitosis, the cell cycle, and other transcriptional programs. Their antitumor effects therefore cannot be attributed entirely to OnF inhibition.

### 5.3 Timing and evidentiary limitations of combination with chemotherapy and EGFR–MAPK-targeted therapy

Should OnF-associated interventions enter clinical practice, their more rational current positioning would be as adjunctive or sequential components of standard therapy. Chemotherapy and targeted therapy can reduce the proliferative tumor burden but may also induce or select for low-proliferative, DTP-like, and fetal-like residual cells. The central objective of candidate strategies is to restrict the state transitions, survival, and post-treatment regeneration of these cells rather than merely intensifying the inhibition of proliferation (9, 24, 103).

The EGFR–MAPK pathway represents an important interface between established CRC treatments and cell-state adaptation. Anti-EGFR therapy is indicated for metastatic CRC with selected RAS/BRAF backgrounds, but its efficacy may be limited by bypass activation, MAPK reactivation, and cell-state transitions (59, 115). In addition to restoring proliferative signaling, MAPK can enhance AP-1 activity and stress-responsive transcriptional programs (127). Combining YAP/TEAD or AP-1 inhibition with therapies targeting EGFR, KRAS, or BRAF is mechanistically justified. However, whether the efficacy of these combinations depends on restricting transitions into the OnF state remains to be established using state-specific biomarkers and functional experiments.

In CRC, FGF/FGFR signaling more commonly acts as a stromal-derived or paracrine input into cellular plasticity. FGFR inhibitors have shown limited efficacy in patients with CRC harboring FGF/FGFR alterations, suggesting that selection based solely on mutations, amplification, or ligand expression may be insufficient (128, 129).

The timing of administration may also influence therapeutic efficacy. Continuous, concurrent, and systemic inhibition of YAP/TAZ–TEAD may impair injury repair and barrier restoration in the normal intestine, thereby increasing the risks of diarrhea, inflammation, and defective mucosal healing (15). In principle, short-course, intermittent, local, or response-adaptive regimens may offer a more favorable therapeutic window. However, direct comparative studies are currently lacking.

### 5.4 Differentiation therapy, biomarkers, and dynamic stratification

Differentiation therapy aims to redirect highly plastic CRC cells toward stable, mature lineages rather than relying solely on cytotoxic elimination. By reconstructing intestinal lineage-regulatory networks, the CANDiT framework identified a CDX2-centered state vulnerability and candidate nodes such as PRKAB1 (130). Chemical perturbation of HDAC1/2 can also alter H3K27ac and H3K9ac states, partially release differentiation blockade, and suppress tumor growth (131).

Current evidence for differentiation therapy is derived predominantly from preclinical models, and target specificity, toxicity to normal tissues, and durability of therapeutic efficacy remain to be evaluated. Efficacy assessment should not be limited to the upregulation of differentiation markers but should demonstrate sustained reductions in tumor initiation, metastatic reconstitution, and post-treatment recurrence. For cells that survive in low-proliferative or senescence-like states and may support residual lesions through the senescence-associated secretory phenotype (SASP), combination strategies involving pro-apoptotic agents, BCL-XL inhibition, or senolytic approaches warrant further investigation (132–135). Patient stratification should likewise not rely on individual markers such as YAP, CLU, L1CAM, EMP1, or SOX17, but should simultaneously capture enhancement of fetal reference programs, attenuation of adult intestinal epithelial identity, and the associated regulatory or functional dependencies.

ColoStem and EpiColoStem have shown prognostic value in retrospective cohorts (136), but neither has been validated as a predictive biomarker of benefit from a specific therapy. Their thresholds may also be influenced by the assay platform, tumor purity, stromal proportion, normalization method, and the fetal reference dataset used. Prospective studies should incorporate CMS/CRIS subtypes, MSI status, RAS/BRAF genotype, spatial niches, and paired samples collected before and after treatment.

The OnF state is dynamic, and sampling at a single time point is unlikely to capture state transitions under therapeutic pressure. ctDNA can be used to assess molecular residual disease (MRD) and the risk of recurrence. However, ctDNA primarily reflects residual tumor burden, tumor-derived molecular alterations, and the probability of recurrence; it does not directly measure fetal reference programs, adult intestinal epithelial lineage identity, or OnF-associated functional dependence (136–139). A more rational approach would be to use ctDNA as a longitudinal indicator of disease burden in combination with tissue- or liquid-based OnF assays. Clinical implementation will also require evidence that the corresponding intervention improves outcomes in biomarker-positive patients.

### 5.5 Limitations of current models and measurement platforms

Patient-derived organoids can preserve diverse epithelial genotypes and state transitions but generally lack the *in situ* immune, vascular, neural, microbial, and stromal compartments (10, 113). Culture media may also select for specific Wnt or growth factor dependencies. Mouse models permit lineage tracing and experimental perturbation but differ from human CRC in anatomy, combinations of mutations, immune history, metastatic patterns, and treatment exposure. Findings obtained in any single system therefore require validation in independent models and human samples.

Single-cell RNA sequencing is affected by tissue-dissociation bias, dropout, batch effects, and sequencing depth. Single-cell chromatin assays provide regulatory information, but their interpretation often relies on inference of enhancer function. Spatial transcriptomics preserves spatial information but has limited cellular resolution and may combine epithelial and stromal signals. Fetal reference features may also vary according to species, gestational age, intestinal segment, analytical platform, and computational workflow. A robust OnF classifier should therefore use a locked gene set or model, prespecified thresholds, orthogonal validation, and external cohorts (10, 17, 18, 30, 86, 107–109).

Most current clinical associations are derived from retrospective studies and cannot determine whether the OnF state causes recurrence or merely co-occurs with aggressive tumor subtypes (9, 30, 136). Establishing causality, reproducibility, and treatment dependence will require longitudinal paired sampling, perturbation–rescue experiments, lineage tracing, and biomarker-stratified clinical trials.

## 6 Conclusions and future perspectives

Oncofetal reprogramming is a biologically meaningful and experimentally testable pathway of plasticity in CRC, but it is not a common endpoint of all injury, inflammatory, stromal, or treatment-related signals. A stringent definition of OnF requires consistent similarity to fetal intestinal reference states, attenuation of adult lineage identity, activation of coordinated fetal or regenerative programs at the gene-set level, and orthogonal support from chromatin or functional evidence. When these criteria are not met, individual markers and general plasticity phenotypes should be described as overlapping evidence rather than as direct evidence of OnF.

The strongest direct mechanistic evidence currently available for CRC OnF primarily supports a role for YAP/TAZ–TEAD and AP-1 in the establishment, maintenance, or therapeutic adaptation of the OnF state in selected models with clearly defined fetal references and functional perturbation evidence. Wnt/APC alterations mainly provide a genotype-dependent background, whereas EGFR/FGFR–MAPK, TGF-β, CAF–ECM mechanotransduction, and inflammatory signals usually act as context-dependent regulatory inputs. These inputs should be classified as direct OnF mechanisms only when a specific study demonstrates that they concurrently induce similarity to fetal reference states, attenuation of adult lineage identity, coordinated activation of fetal programs, and orthogonal functional changes. Otherwise, they should be described as overlapping mechanisms, niche associations, or mechanistically plausible inputs, as appropriate. Inflammation-driven dedifferentiation, Notch-dependent progenitor maintenance, EMP, alternative stemness, DTP states, and immune exclusion may intersect with OnF programs but can also arise independently through non-OnF mechanisms. Future studies must simultaneously consider genotype, molecular subtype, tumor location, disease stage, metastatic organ, prior treatment, and sampling time. Findings from a single organoid system, animal model, or spatial region should not be generalized to all CRCs.

Clinical translation should be based on a validated definition of the cell state rather than inferred solely from pathway activity. Future priorities include standardization of fetal reference classifiers, prospective longitudinal cohorts, spatial and functional validation, comparisons of dosing schedules, and biomarker-stratified trials capable of distinguishing prognostic associations from predictive treatment benefit. The therapeutic goal is not to abolish normal regeneration, but to preserve intestinal repair while restricting the capacity of residual malignant cells to repeatedly reconfigure their states, readapt, and drive recurrence.
